# The Biology and Role of Interleukin-32 in Tuberculosis

**DOI:** 10.1155/2018/1535194

**Published:** 2018-10-22

**Authors:** Wu Li, Wanyan Deng, Jianping Xie

**Affiliations:** ^1^Key Laboratory of Regional Characteristic Agricultural Resources, College of Life Sciences, Neijiang Normal University, Neijiang, Sichuan 641100, China; ^2^Key Laboratory of Molecular Biology for Infectious Diseases (Ministry of Education), Institute for Viral Hepatitis, Department of Infectious Diseases, The Second Affiliated Hospital, Chongqing Medical University, Chongqing, China; ^3^Institute of Modern Biopharmaceuticals, State Key Laboratory Breeding Base of Eco-Environment and Bio-Resource of the Three Gorges Area, Key Laboratory of Eco-environments in Three Gorges Reservoir Region, Ministry of Education, School of Life Sciences, Southwest University, Beibei, Chongqing 400715, China

## Abstract

Tuberculosis, caused by *Mycobacterium tuberculosis*, remains a leading cause of morbidity and mortality globally, with nearly 10.4 million new cases of incidence and over 1.7 million deaths annually. Drug-resistant *M. tuberculosis* strains, especially multidrug-resistant or extensively drug-resistant strains, have further intensified the problem associated with tuberculosis control. Host-directed therapy is a promising alternative for tuberculosis control. IL-32 is increasingly recognized as an important host molecule against tuberculosis. In this review, we highlight the proinflammatory properties of IL-32 and the mode of action of IL-32 in mycobacterial infections to inspire the development of novel immunity-based countermeasures and host-directed therapies against tuberculosis.

## 1. Introduction

*Mycobacterium tuberculosis*, the causative agent of tuberculosis (TB), latently infected one-third of the global population. TB is a global public health threat, with 10.4 million new cases and 1.7 million TB-associated deaths reported worldwide in 2016. New classes of effective anti-TB antibiotics are urgently needed [1] largely due to the occurrence of drug-resistant *M. tuberculosis*. Six hundred thousand new cases are rifampin resistant, including four hundred and ninety thousand patients exhibiting multidrug-resistant infection (http://www.who.int/tb/publications/global_report/en/). Host-directed therapy is a promising direction for the treatment of TB. Interleukin-32 (IL-32), originally called NK cell transcript 4 (NK4), can be produced by human NK and T cells stimulated with IL-2 [2]. IL-32 is a pleiotropic cytokine that can induce proinflammatory cytokines such as TNF-*α* and IL-1*β* via activation of NF-*κ*B and p38 MAPK signaling [3]. IL-32 is primarily found only in primates [3, 4]; in humans, this gene is located on chromosome 16p13.3 and consists of eight exons [3, 5]. The presence of IL-32 mRNA in both immune and nonimmune tissues and cells, including NK cells, T cells, dendritic cells, endothelial cells, and epithelial cells [6, 7], suggests that this gene has multiple functions [7–10], such as inflammatory response [3], apoptosis [11], cell death [12], differentiation [8, 9], and in the pathogenesis of inflammatory disorders, including rheumatoid arthritis [13, 14], allergic rhinitis [15, 16], neuromyelitis optica [17], inflammatory bowel disease [18], chronic rhinosinusitis [19], osteoporosis [20], atherosclerosis [21], cardiovascular diseases [22], pulmonary diseases [23], Crohn's disease [24], Behçet's disease [25], hidradenitis suppurativa [26], cancer [27], and myeloid leukemia [28]. IL-32, as a proinflammatory cytokine, has been extensively studied [29], and the mechanisms of action and functions of IL-32 during bacterial and viral infection as well as in cancer have been reviewed [30–32]. IL-32 plays protective roles in multiple infectious diseases, such as HIV [33–35], influenza [36], cytomegalovirus [37], HBV [38, 39], *Leishmania braziliensis* [40, 41], *Mycobacterium avium* [42], and *M. tuberculosis* [43, 44] infection. In this review, we highlight the immunomodulatory effects and signaling pathways of IL-32 during mycobacterial infection.

## 2. The Isoforms and Secretion of IL-32

Many cytokines have multiple splicing isoforms. IL-17, IL-15, and vascular endothelial growth factor (VEGF) as well as IL-32 possess differently spliced isoforms. IL-15 has two alternatively spliced isoforms with identical biological properties but distinct modes of regulation and expression patterns [45]. There are nine alternatively spliced isoforms of IL-32 in the GenBank database (https://www.ncbi.nlm.nih.gov/genbank/), namely, IL-32*α*, IL-32*β*, IL-32*γ*, IL-32*δ*, IL-32*ε*, IL-32*ζ*, IL-32*η*, IL-32*θ*, and IL-32s, generated by alternative mRNA splicing [46]. These isoforms interact with each other to control their biological activities [46]. IL-32 isoforms IL-32*δ* and IL-32*β* can interact. IL-32*δ* interacts with IL-32*β* and inhibits IL-32*β*-induced production of IL-10 [47]. The sequence of IL-32*β* is similar to that of IL-32*γ* which is spliced into IL-32*β* in different cell lines, such as THP-1, HeLa, and human synovial fibroblast cells [48, 49]. IL-32*α* is frequently observed in the cytosol but not in the culture supernatants of epithelial cells, including primary keratinocytes, intestinal epithelial cell lines, and colonic subepithelial myofibroblasts [18, 50, 51]. IL-32*α* specifically binds to proteinase-3 with high affinity, and this binding is independent of enzyme activity [52]. IL-32*α* has been reported to interact with PKC*ε* and STAT3 [53] and with focal adhesion kinase 1 (FAK1) and integrins [54]. IL-32*β* and IL-32*γ* can induce caspase-8- and caspase-3-dependent apoptosis [54, 55]. IL-32*β* interacts with C/EBP*α* and PKC*δ*, culminating in increased IL-10 production [56]. IL-32*γ*, without exon deletions, is the most active isoform [46, 57].

The secretion of IL-32 isoforms remains to be investigated. IL-32*γ* possesses an N-terminal hydrophobic signal peptide, which is a typical feature of secreted cytokines. IL-32 is expressed in peripheral blood mononuclear cells (PBMCs) by LPS stimulation or *M. tuberculosis* infection, instead of *Staphylococcus aureus* and *Candida albicans* [58]. The IL-32*α* isoform was detected as an intracellular fraction, whereas the IL-32*β* isoform was found in the cell culture supernatant of Cos7 cells under transient transfection [3]. However, when performing transient transfection of IL-32*β* into bovine aortic vascular endothelial cells (BAVECs), IL-32*β* was found mainly in the cytosol and localized in the endoplasmic reticulum [6]. In addition, IL-32*β* was detected in the supernatant derived from the cytoplasm of apoptotic T cells but not secreted in anti-CD3 antibody-activated human T cells [12]. However, IL-32 can bind to the RGD motif of integrin, and IL-32 isoforms contain predicted tyrosine sulfation sites, which are prevalent in secreted proteins [2, 5, 59]. In HT-29 cells stimulated with TNF-*α* and IFN-*γ*, IL-32 was associated with membrane vesicles, and the release of IL-32 depended on exosome-like vesicle release mechanisms [60]. Therefore, IL-32 may be secreted via a nonclassical protein secretion pathway, similar to IL-33 and HMGB1, without typical signal peptides and are released via ER/Golgi-independent means [60, 61].

## 3. The Cellular Source and Expression of IL-32

IL-32 does not share homology with known cytokines. IL-32 expression has been detected in multiple human tissues and organs, including spleen, thymus, leukocytes, lungs, heart, placenta, liver, muscle, kidneys, pancreas, prostate, small intestine, colon, and brain [3]. The IL-32 mRNA is highly expressed in immune cells, and IL-32 expression has also been detected in nonimmune tissues and cells [6, 55, 62]. NK cells [2, 3, 63], monocytes/macrophages [3, 62, 64], dendritic cells (DCs) from PBMCs [58, 62, 65], neutrophils [66], T lymphocytes [62], epithelial cells [67], endothelial cells [68], fibroblasts [69], and hepatocytes [64] can express IL-32. IL-32 is also expressed and released in both cancer and noncancer cell lines, including the HepG2 human cancer cell line [3, 70], A549 cells [71, 72], pancreatic cancer cell lines such as MIA PaCa-2, PANC-1, and BxPC-3 [73, 74], the human hepatoma cell line Huh-7.5 [64], cervical cancer cells and tissues [75], the HEK293T cell line [34, 57], the HT-29 human colon cell line [60], the human colon neuroendocrine LCC-18 cell line [34], human colonic subepithelial myofibroblasts [51], human primary keratinocytes [50], synovial cells and fibroblast-like synoviocytes (FLS) [14, 69], and the marrow stromal cell lines HS-5 and HS-27A [76].

Four major isoforms (IL-32*α*, IL-32*β*, IL-32*γ*, and IL-32*δ*) were found in IL-2-stimulated human NK cells [3]. IL-32*β*, IL-32*ε*, and IL-32*ζ* were isolated from activated T cells [12], and IL-32s expression was first observed in Jurkat human leukemia T cells [70]. IL-32*ε*, IL-32*ζ*, IL-32*θ*, and IL-32s are also found in T cells, and the IL-32*β* isoform is mainly expressed in activated T cells [2, 12, 46]. IL-32*θ* and IL-32s were identified from monocyte-derived dendritic cells purified from human PBMCs and Jurkat T cells via 5′ RACE [46]. The function of different IL-32 isoforms in different cell types was summarized in Table 1. IL-32 mRNA levels increased after stimulation with Con A and monoclonal antibodies against CD3 and CD28 [62]. TNF-*α* reciprocally induced the expression of IL-32 mRNA in monocyte-derived dendritic cells, T cells, and synovial fibroblasts [62]. Intracellular IL-32 is constitutively expressed in human umbilical vein endothelial cells (HUVECs). The IL-32*α* and IL-32*γ* isoforms are the most prominently expressed IL-32 mRNAs in unstimulated endothelial cells [6, 60, 68, 77], while TNF-*α* and IL-1*β* induced the expression of IL-32*β* in endothelial cells [4]. Studies have shown that GM-CSF induces the expression of the IL-32*α*, IL-32*β*, IL-32*γ*, and IL-32*δ* isoforms in a caspase-1-dependent manner in eosinophils [15, 16]. Synovial fibroblasts isolated from patients with rheumatoid arthritis express IL-32*γ* after stimulation with IL-1*β* and TNF-*α* [48]. TNF-*α* can also promote the expression of the IL-32*α*, IL-32*β*, IL-32*δ*, and IL-32*γ* isoforms by activating the Syk/PKC*δ*/JNK/c-Jun signaling pathway [69]. The cell or tissue-specific expression patterns and functions of each isoform of IL-32 remain to be determined.

## 4. The Function of IL-32 in the Activation of Signaling Pathways

**Figure 1:**
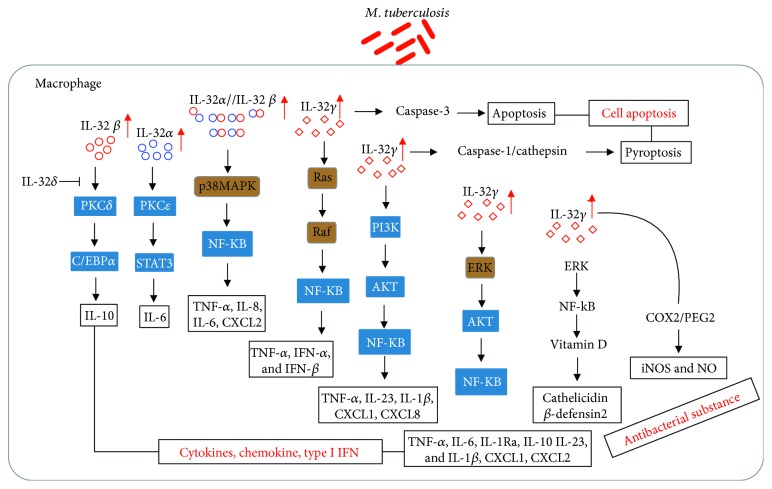
Endogenous IL-32-induced signaling pathway activation in macrophages and the potential roles of this pathway in *M. tuberculosis* infection.

Although proinflammatory activities are key features of IL-32 and are enhanced by the different IL-32 isoforms, which induce the expression of cytokines such as TNF-*α* [3], IL-1*β* [87], IL-6 [53], IL-8 [88], and COX-2 [75], the mechanism of IL-32-based signaling remains unknown. The potential signaling pathways of macrophages induced by IL-32 are summarized in Figure 1. IL-32*α*, IL-32*β*, and IL-32*γ* are the main isoforms of IL-32 and have been shown to enhance the inflammatory response, suggesting that IL-32 can mediate diverse responses by interacting with different signaling molecules [53, 54, 56]. Intracellular IL-32*α* interacts with PKC*ε* and STAT3, leading to phosphorylation of STAT3 and induction of IL-6 production after PMA stimulation [53]. Induction of TNF-*α* by IL-32*α* is mediated by phosphorylation of inhibitor kappaB (IkB) and ERK1/2 [89], NF-*κ*B activation, and p38 MAPK phosphorylation in macrophage cell lines such as THP-1 and RAW264.7 [3]. Both IL-32*α* and IL-32*β* induce the expression of TNF-*α*, IL-8, and CXCL2 in THP-1 and RAW264.7 cells [3, 62] and induce the expression of TNF-*α* and CXCL2 in peritoneal murine macrophages [57]. Treatment of THP-1 cells with IL-32*γ* induced TNF-*α*, IL-6, IL-1*β*, and IL-8 expression via activation of the p38, caspase-1, and NF-*κ*B pathways [16]. In addition, IL-32*γ*-stimulated monocytes and macrophages, such as THP-1-derived macrophages and monocyte-derived macrophages, induce the expression of TNF-*α*, IL-1*β*, IL-6, CXCL1, and CXCL2 along with IL-1Ra and IL-10 via the ERK1/2 and Akt signaling pathways [80]. Moreover, IL-32*γ* triggers the production of TNF-*α*, IL-1*β*, IL-23, CXCL1, and CXCL8 via the PI3K/Akt/P300/NF-*κ*B signaling pathway [81]. PR3 cleaves IL-32*α* and increases the activity of IL-32, which subsequently activates PAR2 and triggers the TRIF and Ras/Raf pathways, resulting in increased type I IFN (IFN-*α* and IFN-*β*) and TNF-*α* production [90]. However, IL-32 isoforms can reduce cellular inflammation [47, 65]. IL-32*δ* inhibits the binding of IL-32*β* to PKC*δ*, resulting in decreased IL-10 production [47]. In monocyte-derived DCs and human macrophages, endogenous IL-32*β* promotes IL-10 expression, resulting in decreased expression of proinflammatory cytokines, such as IL-12, TNF-*α*, and IL-1*β* [65]. IL-32*β* promotes IL-10 production via interaction with PKC*δ*, which phosphorylates C/EBPa, an inhibitor that binds to the IL-10 promoter [56]. Moreover, low-severity arthritis was observed in a human IL-32*β* transgenic mouse model [91]. In summary, IL-32 regulates the expression of inflammatory cytokines.

## 5. IL-32 Regulates the Expression of MicroRNAs

IL-32 isoforms were shown to induce inflammation by regulating the expression of microRNAs [20, 37, 92, 93]. The expression of IL-32 is activated by human cytomegalovirus infection and functionally downregulated by hcmv-miR-UL112-1 [37]. MiR-23b-3p directly targets and induces the expression of PTEN, resulting in reduction in PI3-kinase, total Akt, and IL-32 levels [93]. IL-32*α* promotes the expression of the atheroprotective-associated genes Timp3 and Reck by downregulating the Rprd2-Dgcr8/Ddx5-Dicer1 biogenesis axis downstream of microRNA-205 [92]. Overexpression of human IL-32*γ* in transgenic mice led to increased bone formation, reduced bone loss with advancing age, and high osteogenic capacity of osteoblasts by upregulation of microRNA-29*α* [20]. Therefore, IL-32 is a novel protective cytokine that acts against mycobacterial infection. Elucidating the complex interactions between the IL-32 isoforms, microRNA-based regulation of the isoforms and the function of IL-32 will provide novel insight into the novel mechanism of the protective roles of IL-32 in multiple diseases.

## 6. The Function of IL-32 in Mycobacterial Infection

*M. tuberculosis*, the causative agent of human TB, can subvert host immune defenses to promote its own intracellular survival. Infection of human macrophages or PBMCs with *M. tuberculosis* H37Rv induced IL-32 production [11, 58], suggesting a role for IL-32 in the control of *M. tuberculosis* infection. *M. tuberculosis* and *Mycobacterium bovis* induced the release of IL-32 from PBMCs via IFN-*γ*, which was produced after caspase-1-activated IL-18 release [58]. Silencing of endogenous IL-32 in differentiated THP-1 human macrophages significantly decreased TNF-*α*, IL-1*β*, and IL-8 production and simultaneously increased the *M. tuberculosis* burden in infected macrophages [11].

The antimycobacterial effect of IL-32 may be partly due to enhanced cell apoptosis in infected macrophages. IL-32*γ* is a potent inducer of apoptosis; both IL-32*γ* and IL-32*β* can induce caspase-3- and caspase-8-dependent apoptosis [12, 27]. Endogenous IL-32 mediated *M. tuberculosis*-induced apoptosis of macrophages, suggesting that apoptosis of infected macrophages is a mechanism to protect against mycobacterial infection. IL-32*γ* decreased the *M. tuberculosis* burden within macrophages via classic caspase-3-mediated apoptosis [11] and caspase-1- or lysosomal-cathepsin-mediated apoptosis [94]. Our previous study showed that *M. tuberculosis* PE/PPE (Pro(P)-Glu(E) and Pro(P)-Pro(P)-Glu(E)) family antigen PPE32 induced ER-stress-mediated cell apoptosis via the stimulation of IL-32 production [95]. In addition, IL-32 serves as a mediator of IFN*γ*-vitamin D-related antimicrobial activity and a marker for latent TB infection (LTBI), as determined via the mining of TB transcriptomic datasets [96]. IL-32*γ* was also found to be associated with the vitamin D antimicrobial pathway in human macrophages [84]. IFN-*γ*-induced IL-32*γ* increases the expression of the vitamin D receptor, leading to the expression of cathelicidin and *β*-defensin 2 (DEFB4), which are potent antimicrobial peptides that act against intracellular infection in macrophages [84]. IFN-*γ* treatment activates the production of NO in macrophages, which is the main microbicidal molecule involved in the control of *M. tuberculosis* infection [97]. Human THP-1 cells express iNOS and produce NO after differentiation into macrophages by treatment with IL-32*γ* [98]. The production of reactive oxygen species (ROS) is required to induce the microbicidal activity mediated by vitamin D and cathelicidin, and cathelicidin enhances the production of ROS and proinflammatory cytokines, such as TNF-*α*, IL-8, and IL-6 [99]. *M. tuberculosis*-induced GM-CSF can promote NO production and phagolysosomal fusion against *M. tuberculosis* infection [100, 101]. GM-CSF might kill intracellular *M. tuberculosis* via induction of IL-32 as GM-CSF increases the expression of IL-32 in other cell types [15, 16]. In summary, IL-32*γ* is a protective molecule that enhances the microbicidal activity of macrophages against *M. tuberculosis* via increased apoptosis and pyroptosis, and antimicrobial peptides induced by vitamin D and GM-CSF are involved in protection against *M. tuberculosis* infection (Figure 1).

IL-32, lacking sequence homology with known cytokine families, is a novel proinflammatory cytokine [3]. The expression of IL-32 was increased in patients with *M. avium* infection [42]. IL-32*γ* significantly reduced the intracellular survival of *M. avium* in human monocyte-derived macrophages [42]. Moreover, the expression of endogenous IL-32 and NOD2 was increased in patients with the restrictive tuberculoid form of leprosy, which is caused by *Mycobacterium leprae* infection [102], suggesting that both NOD2 and IL-32 are associated with leprosy. IL-32 expression was increased in surgically resected lungs of active TB patients, particularly in airway epithelial cells and granuloma macrophages [43], suggesting a protective role of IL-32 against *in vivo M. tuberculosis* infection. However, there was a decrease in the protective response of IL-32*γ* against *M. tuberculosis* at later time points of infection as IL-32*γ* mRNA is spliced into IL-32*β*, leading to increased levels of IL-10-expressing macrophages or DCs in the lungs [43].

## Figures and Tables

**Table 1:** The function of IL-32 isoforms in different cell type.

Cell type	IL-32 isoform	Targets	Function	Reference
U937 and monocyte-derived DCs	IL-32*β*	Increase in IL-10 production	Anti-inflammatory effects	[65]
Tumor cells	IL-32*β*	Decrease IL-1*β*, IL-6, TNF-*α*, and increase IL-10 production	Tumor growth	[78]
Myeloid cells and U937 cells	IL-32*β*	Increase in IL-10 production	Anti-inflammatory effects	[56]
Eosinophils	IL-32*γ*	Induces production of IL-6, TNF-*α*, IL-8, and VEGF	Inflammation of allergic rhinitis	[15]
Eosinophils	IL-32*γ*	Induces IL-1*β*, TNF-*α*, CXCL8, CCL3, CCL4, CD18, and ICAM-1	Interacts with NOD1 or NOD2; PR3 activation	[79]
Monocytes or monocyte-derived macrophages	IL-32*γ*	TNF-a, IL-1b, IL-6, GROa/CXCL1, and MCP-1/CCL2, IL-10, and IL-1ra	Activation of ERK1/2, Akt, and Fyn signaling pathways	[80]
PBMC	IL-32*α*/*β*	TNF-*α*, IL-6	—	[57]
Murine macrophage	IL-32*α*/*β*	TNF-*α*, CXCL2	—	
THP-1 and RAW264.7	IL-32*α*/*β*	TNF-*α*, IL-8, and, CXCL2	—	[3, 62]
THP-1 cells	IL-32*γ*	Induces TNF-a, IL-1b, IL-8, and IL-6	Activation of p38, caspase-1 and NF-*κ*B pathways	[16]
THP-1 cells	IL-32*γ*	TNF-*α*, IL-23, CXCL1, CXCL8, and IL-1*β*	PI3K/Akt/P300/NF-*κ*B signaling pathways	[81]
Endothelial cells	IL-32*α*/*β*/*ε*	ICAM-1, IL-1*α*, IL-8, and IL-6	Vascular inflammation	[68]
PBMC/precursors	IL-32*α*	Activates Akt, JNK, ERK1/2, and NF-*κ*B pathways	Cell differentiation	[10]
Murine DC	IL-32*γ*	Suppresses the production of CCL5	Driving acquired immunity	[82]
Murine bone marrow–derived DCs	IL-32*γ*	IL-6 and IL-12	Driving acquired immunity	[83]
PBMCs, CD4^+^ T cells, CD163^+^ macrophages, Treg cells, and DCs	IL-32*γ*	IDO and ILT4	Immunosuppression	[35]
Monocyte-derived macrophages	IL-32*γ*	Induce cathelicidin and *β*-defensin 2 (DEFB4)	Microbicidal activity	[84]
PBMC	IL-32*γ*	IFN*λ*1	Antiviral activity	[85]
T cells, epithelial cells, THP-1, and tumor cells	IL-32*γ*/*β*	Caspase-3, Caspase-8	Cell apoptosis	[12, 27]
THP-1 cells	IL-32*θ*	Suppresses the production of CCL5	Modulators of inflammation	[86]
THP-1 cells	IL-32*θ*	Decreases TNF-*α*	p38 and NF-*κ*B signaling pathways	[28]
